# Vanishing bone metastases: a pitfall in contrast-enhanced CT in patients with venous thrombosis

**DOI:** 10.1259/bjrcr.20150149

**Published:** 2015-07-03

**Authors:** R Pruente, CD Strickland, PJ Koo

**Affiliations:** Department of Radiology, School of Medicine, University of Colorado, Aurora, CO, USA

## Abstract

Enhancement patterns of visceral venous collaterals are well documented in cases of superior vena cava obstruction. Only recently has intraosseous venous collateral enhancement been described. We describe an unusual case of vertebral marrow enhancement in the lower thoracic spine related to venous collateral circulation caused by an incidental hemiazygos thrombus. Misinterpretation of this finding can lead to the erroneous interpretation of sclerotic bone metastases.

## Case presentation

A 66-year-old female with a history of breast and lung cancer, both treated and in remission, presented with new onset of generalized abdominal pain. Physical examination and laboratory work-up were unrevealing as to a potential cause.

## Investigations

**Figure 1.**
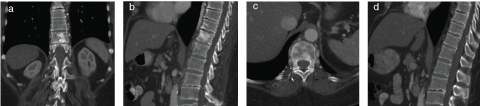
(a–d) A 66-year-old female with a history of lung and breast cancer presents with new onset abdominal pain. Contrast-enhanced CT scan of the abdomen and pelvis (a–c) reveals abnormal high attenuation within the lower thoracic vertebrae. Comparison was made with a prior study performed in 2011 (d), which showed no bone abnormality at that time. This was interpreted as new osseous metastatic disease.

A contrast-enhanced CT (CECT) of the abdomen and pelvis was performed with series obtained in both arterial and delayed phases that failed to reveal an abdominal or pelvic source of the pain. Comparison with a CECT performed 2 years prior, however, revealed new areas of high attenuation in the T11 and T12 vertebral bodies and posterior elements (Figure 1). This was initially attributed to recurrence of disease with new osseous involvement.

**Figure 2.**
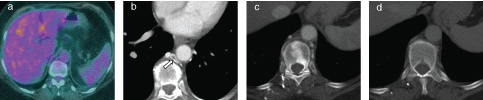
(a–d) Non-contrast positron emission tomography CT centred at the site of previous abnormal vertebral body attenuation demonstrates no 18-fludeoxyglucose-avid metastatic disease with the abnormal attenuation in question no longer apparent (a). Review of the prior contrast-enhanced CT scan in question (b–d) revealed subtle thrombus in the hemiazygos vein (arrow) with much of the abnormal attenuation centred in the basivertebral veins (c). On delayed phase, the abnormal attenuation has resolved (d).

Given the potential of tumour recurrence, a positron emission tomography CT scan was performed without contrast to evaluate the lesion and to assess for other sites of disease. The study revealed normal-appearing thoracic vertebrae without evidence of 18-fludeoxyglucose-avid metastatic disease. On review of the prior CECT in question, it was noted that the majority of the high density areas were centred in the region of the basivertebral veins. In retrospect, this finding was present on arterial phase images and absent on the delayed phase images that prompted a search for the potential cause of this atypical enhancement pattern. Careful review near the source of marrow enhancement revealed incidental thrombus in the hemiazygos vein (Figure 2). It was concluded that thrombus in the hemiazygos vein had caused retrograde opacification of the vertebral venous plexus and its anastomotic capillary sites, which mimicked sclerotic bone metastases.

## Differential diagnosis

The leading diagnosis for a new finding of high attenuation centred in the vertebral body in a patient with known cancer is osteoblastic metastatic disease. Only after careful review was intraosseous venous collateral enhancement considered. Other potential causes of sclerotic bone lesions, including entities such as enostosis, osteoid osteoma or chronic osteomyelitis, were felt less likely given the interval appearance and imaging characteristics.

## Treatment

Following the detection of venous thrombosis as explanation for the abnormal bone findings, the patient was treated with anticoagulation for 6 weeks without complications.

## Outcome and follow-up

To our knowledge, incidental thrombus in the hemiazygos vein is an unreported finding with scarce literature as to its imaging appearance and appropriate management. It was felt the clot was an unlikely cause of the patient’s abdominal pain and was instead an incidental finding. Clinical work-up for a potential source for the clot was unrevealing. The patient was anticoagulated for 6 weeks, with repeat CECT showing resolution of both the hemiazygos thrombus and unusual vertebral body enhancement.

## Discussion

**Figure 3.**
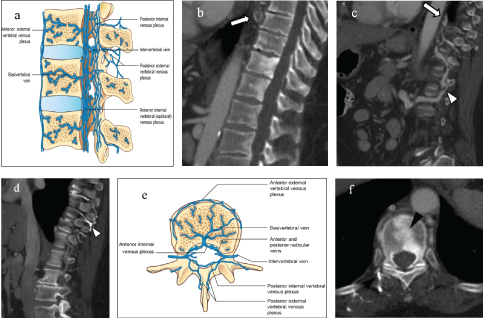
(a–f) Anatomy of the vertebral venous plexus with CT correlation. Sagittal images (a–d) reveal the complexity of the vertebral venous network with numerous internal collaterals bridging the anterior and posterior external venous plexus. These collaterals are usually occult on imaging but can become distended (white arrowhead) by occlusive thrombus (arrow) or venous narrowing upstream. Axial images (e,f) highlight the reflux of contrast through the basivertebral venous system and into the external vertebral venous plexus (black arrowhead). (a,e) As originally depicted by Netter illustration and re-drawn with permission of Elsevier, Inc. All rights reserved. www.netterimages.com

Although unusual, enhancement of venous collateral pathways has been reported, primarily in cases of superior vena cava (SVC) obstruction.^1-4^ The enhancement occurs through 4 main established pathways that can be divided into superficial and deep systems that communicate with one another. The deep pathway includes the (1) azygos and hemiazygos veins, (2) internal and lateral thoracic veins, and (3) vertebral venous plexus (Figure 3). The superficial pathway includes the (4) thoraco-Jepigastric and intercostal veins.^5–9^

Predictably, the pattern of venous collateral enhancement will depend on the site of venous obstruction as well as variations in venous anatomy and study technique. Atypical hepatic and pericardial enhancement related to collateral reflux has been described in cases of symptomatic obstruction of the SVC.^3,4^ Only recently has intraosseous venous collateral enhancement been characterized. As far as we know, there are only five existing reports and one retrospective review to date.^10-15^ In the majority of these cases, the marrow reflux was centred in the lower cervical or upper thoracic spine related to obstruction at the SVC or near the SVC/innominate vein confluence. To the best of our knowledge, our case is the only reported case of vertebral marrow reflux in the lower thoracic spine related to incidental hemiazygos clot.

Little is known about thrombus in the hemiazygos vein with respect to its imaging appearance. In our case, the initial finding was only identified after a careful search for the cause of atypical marrow enhancement. It was unclear from our study whether the hemiazygos clot was actually fully or near fully occlusive—only that it had elevated venous pressure enough to cause retrograde opacification through the vertebral venous plexus, which was absent on delayed phase. In retrospect, change in the vertebral body appearance on different phase images highlights the need to review potential pathology on all provided phases.

With respect to managing hemiazygos vein thrombosis, literature review reveals scarce information. Work-up for a potential source of the thrombus included a negative sonography and coagulation profile. Given the small clot burden and lack of a potential source, our patient had a CECT performed following 6 weeks of anticoagulation that showed resolution of the thrombus and absence of the previously demonstrated vertebral body enhancement. No additional treatment was performed as it was felt the clot was an incidental finding and an unlikely source of the patient’s abdominal pain.

## Learning points

1. The case presented illustrates a benign entity related to contrast filling the vertebral venous plexus in a retrograde manner owing to thrombus in the hemiazygos vein.
2. Venous collateral enhancement depends on the site of obstruction with previous reports illustrating collateral enhancement in cases of SVC obstruction, typically by an obstructing mass. These patients may present with findings of SVC syndrome, including facial oedema, upper extremity venous congestion, dyspnoea and headache.
3. Little is known about hemiazygos vein thrombosis with regards to its imaging appearance and management. Our patient presented with abdominal pain that was felt to be unrelated to the clot and was anticoagulated for 6 weeks without complication.
4. Whenever there is new or unusual attenuation within a vertebral body, findings on all phases provided should be reviewed and contrast reflux through intraosseous venous collaterals considered as a potential pitfall.
